# 5mC-Related lncRNAs as Potential Prognostic Biomarkers in Colon Adenocarcinoma

**DOI:** 10.3390/biology11020231

**Published:** 2022-02-01

**Authors:** Yinghui Huang, Huiqian Huang, Yong Wang, Hui Liu, Yingdan Huang

**Affiliations:** 1 Key Laboratory of Adolescent Cyberpsychology and Behavior, Ministry of Education, Wuhan 430056, China; yhhuang@ccnu.edu.cn; 2 School of Psychology, Central China Normal University, Wuhan 430056, China; 3 National Research Center of Cultural Industries, Central China Normal University, Wuhan 430056, China; hqhuang@mails.ccnu.edu.cn; 4 Department of Medical Oncology, The First Affiliated Hospital of Nanchang University, 17 YongwaiZhengRoad, Nanchang 330209, China; 361439920025@email.ncu.edu.cn; 5 Department of Lymphoma Medicine, Hubei Cancer Hospital, Tongji Medical College, Huazhong University of Science and Technology, Wuhan 430079, China

**Keywords:** 5-methylcytosine, colon adenocarcinoma, lncRNA signature, prognosis, immunotherapy

## Abstract

**Simple Summary:**

To identify the prognostic significance of 5mC-related lncRNAs in colon adenocarcinoma (COAD), we examined the expression levels and mutations of 21 5mC-regulated genes of COAD in TCGA. We also identified lncRNAs associated with 5mC regulatory genes using Pearson correlation analysis. After the least absolute shrinkage and selection operator (Lasso) Cox regression, the risk signature of 4 5mC-related lncRNAs was selected. Next, the risk signature’s predictive efficacy was proven. Moreover, the biological mechanism and potential immunotherapeutic response of this risk signature were identified. Collectively, we constructed the 5mC-related lncRNA risk signature, which could provide a novel prognostic prediction of COAD patients.

**Abstract:**

Globally, colon adenocarcinoma (COAD) is one of the most frequent types of malignant tumors. About 40~50% of patients with advanced colon adenocarcinoma die from recurrence and metastasis. Long non-coding RNAs (lncRNAs) and 5-methylcytosine (5mC) regulatory genes have been demonstrated to involve in the progression and prognosis of COAD. The goal of this study was to explore the biological characteristics and potential predictive value of 5mC-related lncRNA signature in COAD. In this research, The Cancer Genome Atlas (TCGA) was utilized to obtain the expression of genes and somatic mutations in COAD, and Pearson correlation analysis was used to select lncRNAs involved in 5mC-regulated genes. Furthermore, we applied univariate Cox regression and Lasso Cox regression to construct 5mC-related lncRNA signature. Then Kaplan–Meier survival analysis, principal components analysis (PCA), receiver operating characteristic (ROC) curve, and a nomogram were performed to estimate the prognostic effect of the risk signature. GSEA was utilized to predict downstream access of the risk signature. Finally, the immune characteristics and immunotherapeutic signatures targeting this risk signature were analyzed. In the results, we obtained 1652 5mC-related lncRNAs by Pearson correlation analysis in the TCGA database. Next, we selected a risk signature that comprised 4 5mC-related lncRNAs by univariate and Lasso Cox regression. The prognostic value of the risk signature was proven. Finally, the biological mechanism and potential immunotherapeutic response of the risk signature were identified. Collectively, we constructed the 5mC-related lncRNA risk signature, which could provide a novel prognostic prediction of COAD patients.

## 1. Introduction

Colon adenocarcinoma (COAD) is the most commonly diagnosed colorectal cancer [1]. The main treatments for COAD include surgery, chemotherapy, radiation therapy, immunotherapy, etc. With the continuous development of treatment, the overall prognostic efficacy of COAD patients has improved dramatically [2,3]. Chemotherapy can be performed as adjuvant therapy following surgery or neoadjuvant therapy before surgery in advanced COAD patients to decrease the tumor [4]. Nevertheless, about 40~50% of advanced COAD patients die from recurrence and metastasis of the disease [5]. Several prognostic signatures have been created to predict drug sensitivity and prognostic efficacy in COAD attributing to the development of RNA sequencing technologies [6,7]. However, no predictive signature combining 5-methylcytosine (5mC)-related long non-coding RNA (lncRNA) and clinical data has been available for COAD patients to use. Hence, there is an urgent need to construct a novel predictive signature for COAD patients that can potentially identify new treatment targets and prognostic markers.

The modification of 5mC is a dynamic reversible process, which comprises methyltransferase (writers), signal transducers (readers), and demethylase (erasers) [8]. In most mammals, CpG islands (CGIs) are a segment of DNA sequence enriched in CpG dinucleotides in the promoter of genes, and the occurrence of 5mC on CGIs is commonly related to the repression of gene expression [9]. Researchers discovered that 5mC regulatory proteins play a critical role in a range of cellular biological processes by modifying DNA methylation [10], such as regulation of gene expression, centriole stabilization, and silencing of retroelements [11,12]. Meanwhile, DNA methylation typically changes in cancer, with hypomethylation occurring in oncogenes and hypermethylation occurring in tumor suppressor gene-regulated regions [13,14]. A mounting body of evidence suggests that downregulation of DNA methyltransferase 1 (Dnmt1) expression would lead to increased mutation rates and cancer development [15]. DNA methylation deficiency is also associated with abnormal oncogene expression [15,16], such as MYC, leading to dysregulation of cellular pathways and tumor development [17]. The eleventh translocase enzyme (TET1, TET2, and TET3) catalyzes the oxidation of 5mC, contributing to the demethylation process. TET1 inhibition is correlated with increased immune marker expression and immune cell infiltration of cancers, including melanoma, lung cancer, and thyroid cancer [18]. Recent research has shown that TET1 can suppress colon tumor progression by inhibiting WNT pathway mediators [19]. Thus, further research on the function of DNA methylation in COAD is critical in identifying novel prognostic indicators and therapeutic targets for malignancies.

Long non-coding RNAs (lncRNAs) are one of a subclass of non-coding RNAs (ncRNAs) that include more than 200 nucleotides [20,21]. An increasing number of studies revealed that lncRNAs can affect the survival, proliferation, migration, and other biological functions of cancer cells by modulating gene expression [22,23,24]. For instance, studies found that dysregulated lncRNAs are well associated with clinical staging and progression of individuals with tongue squamous cell carcinoma [25]. Similarly, it has been shown that LncRNA-ATB triggered by TGF is related to a grim outcome in patients with advanced hepatocellular carcinoma [26]. In addition, exosomal lncRNAs serve as diagnostic and prognostic biomarkers [27]. Lim et al. found that LncRNAH19 is a tumor-promoting factor that promotes tumor growth via EMT. Exosomes carrying H19 can be recruited into breast cancer cells, thereby mediating the cells’ resistance to DOX [28,29]. Furthermore, recent studies pointed out that 5-mC can regulate lncRNA transcript levels and could be used as a new biomarker for the clinical prognosis of malignancies [30,31]. For instance, methylation-regulated LINC00574 could be used as a biomarker for bladder cancer prognosis [32]. Despite 5-hydroxymethylcytosine being required for regulating lncRNA transcription in colorectal cancer [30], no 5mC-related lncRNA signature is involved in COAD prognosis. Therefore, it is important to understand the role of 5mC-related lncRNAs in COAD, which may help us to uncover novel indicators and treatment targets for COAD.

In this study, firstly, we obtained the expression profiles of 13,413 lncRNAs and 21 5mC regulatory genes from the TCGA database. Then, we discovered the lncRNAs associated with 5mC utilizing Pearson correlation analysis. We also constructed a 5mC-related lncRNA prognostic signature. Finally, we explored the immunotherapy responses of the risk signature. This result may provide a novel prognostic biomarker and a new perspective for individualized therapy in COAD patients.

## 2. Materials and Methods

### 2.1. Data Obtaining and Correlation Analysis

RNA transcriptome data and associated clinical features on the COAD patients were downloaded from the TCGA database (https://portal.gdc.cancer.gov, accessed on 20 September 2021). Patients without survival information were deleted. The expression levels of all transcripts were measured using fragments per kilobase million (FPKM) and were standardized using a log2-based transformation. In addition, based on human genome annotation datasets, genes were classified as protein-coding genes or lncRNA genes. The somatic mutation data were downloaded from the TCGA database and the copy number variants of TCGA-COAD were collected from the UCSC Xena database. As shown in the previous study [31], the 21 5mC regulators included 3 writers (DNMT1, DNMT3A, DNMT3B), 15 readers (MBD1, MBD2, MBD3, MBD4, MECP2, NEIL1, NTHL1, SMUG1, TDG, UHRF1, UHRF2, UNG, ZBTB33, ZBTB38, ZBTB4), and 3 erasers (TET1, TET2, TET3). Then, we determined the expression of the 21 5mC regulatory genes. The CNV landscape of 21 5mC regulatory genes was visualized by the R package “Rcircos.” Meanwhile, the somatic mutation data were performed by the “maftools” package. We used Pearson correlation analysis to verify the association between 5mC regulatory genes and noncoding RNAs. A total of 1652 5mC-related lncRNAs were selected based on a correlation coefficient more than 0.4 and a *p*-value less than 0.001.

### 2.2. Identification and Construction of Risk Signature

Patients were randomly assigned to a training group and a testing group. The training dataset was performed to generate a signature for 5mC-related lncRNAs, the testing and complete datasets were employed to verify the created model. First, univariate Cox regression analysis was used to identify the prognostic efficacy of lncRNAs associated with 5mC. After that, we conducted Lasso penalized Cox regression analysis to discover the prognostic model in the training dataset, resulting in the establishment of a four 5mC-related lncRNA risk signature. Next, the 5mC-related lncRNA prognostic signature in the training dataset was identified using Lasso Cox regression analysis, and a four 5mC-related lncRNA risk signature was also established. The following algorithm was performed to calculate the score for each COAD patient:

Risk score = coef(lncRNA1) × expr(lncRNA1) + coef(lncRNA2) × expr(lncRNA2) + ⋯ + coef(lncRNAn) × expr(lncRNAn)

where coef represents coefficients, coef(lncRNAn) represents the coefficient of lncRNAs associated with survival, and expr(lncRNAn) denotes lncRNAs expression. 

Finally, we classified the COAD patients into low- and high-risk subgroups depending on the median risk score.

### 2.3. Evaluation and Verification of the 5mC-Related lncRNA Prognosis Signature 

The COAD patients were divided into high-risk and low-risk groups based on their prognostic risk score using the median risk score in the 5mC-related lncRNA prognosis signature. By utilizing R tools “survival”, “survminer”, and “timeROC”, we measured the signature’s accuracy. The Kaplan–Meier survival curve was performed to estimate the variation in surviving rate of high-risk and low-risk subgroups. The “Rtsne” and “ggplot2” tools were applied to achieve efficient dimensionality reduction, pattern recognition, and cluster depiction on the high-dimensional statistics of the four 5mC-related lncRNAs genes. Using the “survival” tool, we conducted univariate and Lasso Cox regression analysis to detect the individual predictive indicators. The R tool “rms” was employed to estimate the prediction accuracy of COAD individuals constructed by a nomogram.

### 2.4. Function Enrichment Analysis and Immunization Score Analysis

We utilized GO functional annotations and KEGG-enriched analysis to comprehend the 5mC regulating genes’ underlying biologic function. Gene set enrichment analysis (GSEA) is a methodology used to compare biological processes across subgroups. Besides, we measured the immune cell expression in two subgroups based on the constructed signature using CIBERSORT.

### 2.5. Cell Culture and Reverse Transcription and Quantitative PCR Analysis (RT-qPCR)

Normal human intestinal epithelial cells (HCM460) and colorectal cancer cells (SW480, HCT116, LOVO) were cultured in Dulbecco’s modified Eagle’s medium supplied with 10% fetal bovine serum, 100 U/mL of penicillin, and 100 μg/mL of streptomycin. Total RNAs were extracted from cells following the manufacturer’s protocol using RNAiso Plus. The PrimeScriptTM RT kit with gDNA Eraser was used to perform reverse transcription on total RNA. RT-qPCR was conducted according to the manufacturer’s protocol using TB GreenTM Premix (Takara, Dalian, China). The primers for RT-qPCR used in this study were as follows. AC008760.1 Forward:AGAGAGGCTGGGAGGAGGTAGAG; AC008760.1 Reverse:GTGGTATCTGAGTGGGTGGCATTG; AC138207.5 Forward:GCGAGGCGACACAGTGATACAG; AC138207.5 Reverse:GCTCAGAGAAGTGAAGTGGCTTGG; AC156455.1 Forward:AGAGCCAGACACTCGTGAAGGG; AC156455.1 Reverse:AAGTCTTGGTCGCACAGGCATTC; ZEB1-AS1 Forward:ACGGTGTCCTTGCTTTGCTTGG; ZEB1-AS1 Reverse:GTGGTGGGGTGGGGTCAATTC.

### 2.6. Statistical Analyses

The analysis data of this experiment were all produced by R 3.6.0. The two-tailed *t*-test was performed to analyze continuous variables between the two subgroups, and the Chi-square test was conducted to evaluate categorical data. The Kaplan–Meier method was used to create the survival curves for the prognostic analysis, and log-rank tests were applied to assess the significance of differences. Associations between risk characteristics and patients’ clinical outcomes were analyzed by Kaplan–Meier survival curves and Cox proportional hazards models. A *p*-value less than 0.05 on both sides was judged significant.

## 3. Results

### 3.1. The Landscape of Genetic Variation of 5mC Regulatory Genes in COAD Patients 

**Figure 1:**
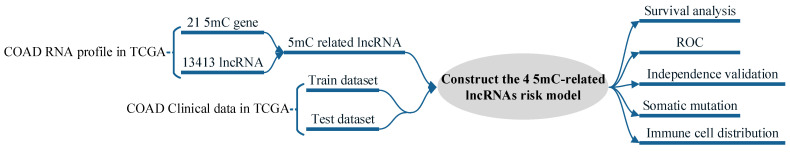
The detailed workflow of the construction of the risk model. Legend: colon adenocarcinoma (COAD); The Cancer Genome Atlas (TCGA); 5-methylcytosine (5mC); long non-coding RNA (lncRNA); receiver operating characteristic (ROC).

**Figure 2:**
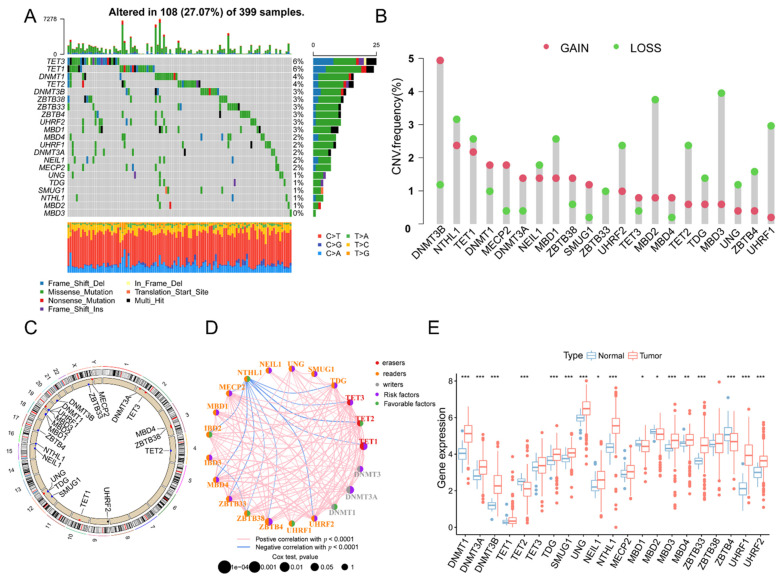
The genetic variation landscape of the 5mC regulatory genes in COAD patients. (**A**) 108 of the 399 COAD patients demonstrated genetic alters in 21 5mC regulators at a frequency of 27.07%. In each column indicates one patient and the numbers to the right represent the incidence of gene mutations. (**B**) the CNV frequency of COAD patients. The column represents the genetic variation, pink dots depict amplification mutations, and green dots depict deletion mutations. (**C**) The location of CNV alteration of 5mC regulatory genes on chromosomes. (**D**) The interaction of 21 5mC regulatory genes. Red, orange, and grey represent erasers, readers, and writers respectively. (**E**) Comparison of the mRNA expression levels of 21 5mC regulatory genes in normal and COAD samples. * *p* < 0.05, ** *p* < 0.01, *** *p* < 0.001.

**Figure A1:**
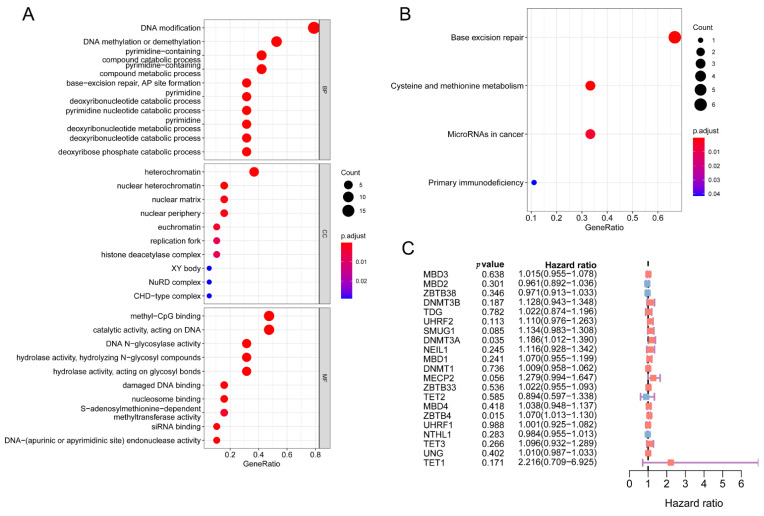
Biological function and prognostic analysis of 21 5mC regulatory genes. (**A**) GO-enrichment analysis of the 21 5mC regulatory genes. (**B**) KEGG enrichment analysis of the 21 5mC regulatory genes. (**C**) Clinical significance of 5mC regulatory genes estimated by univariate Cox regression.

**Figure A2:**
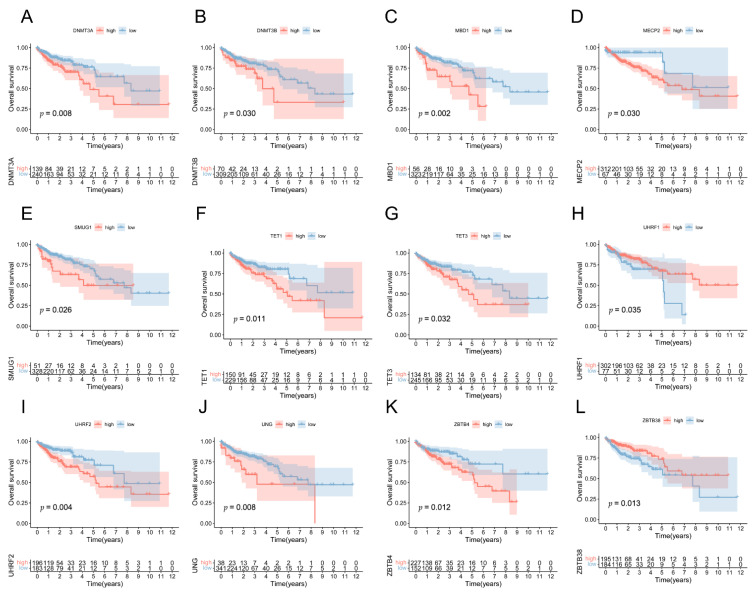
Kaplan–Meier survival curves of DNMT3A (**A**), DNMT3B (**B**), MBD1 (**C**), MECP2 (**D**), SMUG1 (**E**), TET1 (**F**), TET3 (**G**), UHRF1 (**H**), UHRF2 (**I**), UNG (**J**), ZBTB4 (**K**), and ZBTB38 (**L**) in the 21 5mC regulatory genes. The red line represents patients with high expression levels and blue represents patients with low expression.

The detailed workflow of the construction of risk signature and subsequent analysis is shown in Figure 1. In this research, we explored the effects of 21 5mC DNA methylation regulatory genes in COAD patients. As shown in Figure 2A, we evaluated the incidence of somatic mutations in 21 5mC regulatory genes in COAD. We found that 108 of 394 (27.07%) samples carried genetic alterations in 5mC regulators, including amplifications, mutations, and deletions. The most frequent mutation was TET3, followed by TET1, DNMT1, and TET2. Additionally, the analysis of 21 5mC regulators revealed that CNV mutations were prevalent, DNMT3B, DNMT1, MECP2, and DNMT3A were amplifications of CNV. As opposed to MBD3, MBD2, UHRF1, UHRF2, and MDB1, which had prevalent deletions within the CNV (Figure 2B). Meanwhile, the positions of 21 5mC regulators CNV alterations on chromosomes were shown in Figure 2C. On the 5mC regulator network, the interactions between the 21 5mC regulators and their prognostic significance in COAD patients were illustrated comprehensively (Figure 2D). Further investigation illustrated that DNMT1/3A/3B, TDG, SMUG1, UNG, NEIL1, MDB3/4, ZBTB33, and UHRF1/2 were essentially upregulated in tumor samples, while TET2, MBD1, and MBD2 were downregulated in tumor samples (Figure 2E). We conducted GO functional annotations and KEGG enrichment analysis of 21 5mC regulatory genes, revealing that the biological processes were significantly enriched (Figure A1A,B). Then, the association between these 5mC regulatory genes and the survival of COAD sufferers was revealed utilizing Cox regression analysis. The forest plot revealed that DNMT3A and ZBTB4 could be regarded as protecting factors and were remarkably correlated with longer overall survival (Figure A1C). Kaplan–Meier survival analysis showed that 5mC regulatory genes are associated with overall survival in COAD patients (Figure A2A–K). These results demonstrate that 5mC regulatory genes have an essential impact on tumor progression.

### 3.2. Identification of 5mC-Related lncRNAs in COAD Patients 

**Figure 3:**
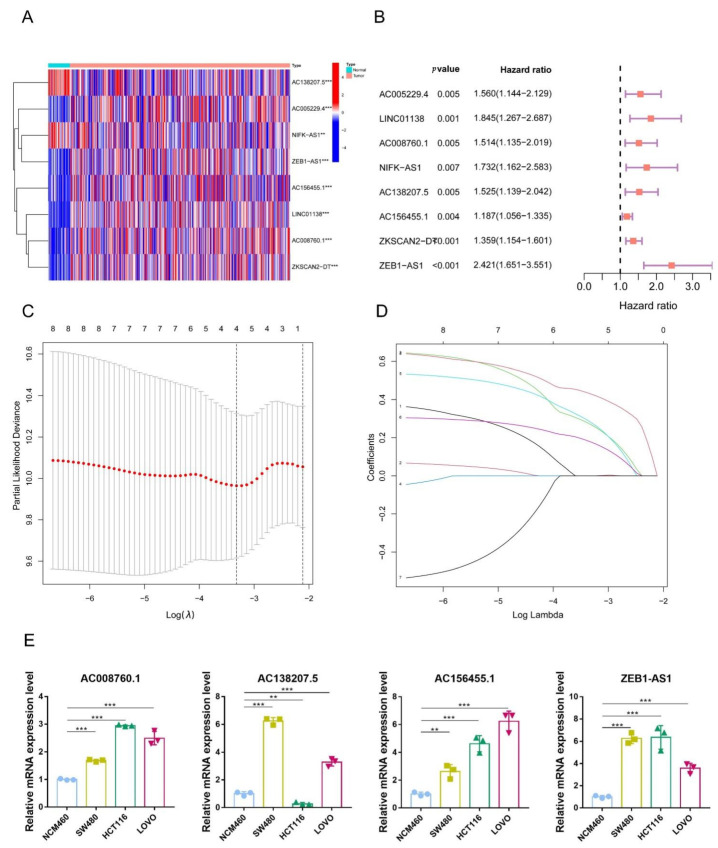
The establishment of the 5mC-related lncRNA signature. (**A**) The expression of selected 5mC-related lncRNAs correlated with survival was depicted by a heatmap utilizing univariate Cox regression. (**B**) The forest map represented the eight 5mC-related lncRNAs that were shown to be significantly associated with prognosis using univariate Cox regression. (**C**) LASSO coefficients for a four 5mC-related lncRNA. (**D**) The selection of the optimal parameters for COAD using the LASSO model. (**E**) Expression levels of AC008760.1, AC138207.5, AC156455.1, and ZEB1-AS1 in normal human intestinal epithelial cells (HCM460) and colorectal cancer cells (SW480, HCT116, LOVO). ** *p* < 0.01, *** *p* < 0.001.

First, owing to the 5mC regulators being critical in the progression of cancer [33,34,35], we recognized 21 5mC regulatory genes by consulting the literature [31,36]. The mRNA expression of 21 5mC regulatory genes and 13413 lncRNAs were downloaded from the TCGA database. We applied the Pearson correlation analysis to characterize potentially 5mC-related lncRNAs. As a result, we observed 611 lncRNAs associated with 5mC regulators with correlation coefficients > 0.4 and a *p*-value < 0.001. Next, we screened 5mC-related prognostic lncRNAs from the 611 5mC-related lncRNAs in the complete dataset utilizing univariate Cox regression analysis. As shown in Figure 3B, 5mC-related lncRNAs were found to be strongly correlated with overall survival in the complete dataset. Then, we depicted the levels of expression of these lncRNAs in normal tissues and tumors by heatmap. As shown in Figure 3A, 2 5mC-related lncRNAs were upregulated in normal tissues, and 6 5mC-related lncRNAs were upregulated in COAD tissues.

### 3.3. Construction of a Signature for 5mC-Related lncRNAs in COAD Patients

A total of 367 COAD patients were randomly assigned to one of two groups: training dataset (*n* = 184) or testing dataset (*n* = 183). Next, Lasso-penalized Cox analysis was used on the 8 prognostic lncRNAs to generate a 5mC-related lncRNA model which contains 4 lncRNAs (Figure 3C,D). Each sample’s risk score was obtained utilizing coefficients of the 4 lncRNAs. (Risk scores=AC008760.1×0.269428721981373+AC138207.5×0.281459243411876+AC156455.1×0.178004647291392+ZEB1−AS1×0.430050179556838). Based on the median prognostic risk value, COAD patients were classified into low-risk and high-risk groups. To investigate the expression of these four lncRNAs in normal colon cells and colon cancer cells, we performed RT-qPCR experiments on normal human intestinal epithelial cells and colorectal cancer cells. The results showed that all four lncRNAs were highly expressed in colorectal cancer cells, except for AC138207.5, which was lowly expressed in HCT116 cells (Figure 3E). Altogether, these results reveal that the four lncRNAs in our signature are probably risk genes in colorectal cancer.

### 3.4. Validation of the 5mC-Related lncRNA Prognosis Signature

**Figure 4:**
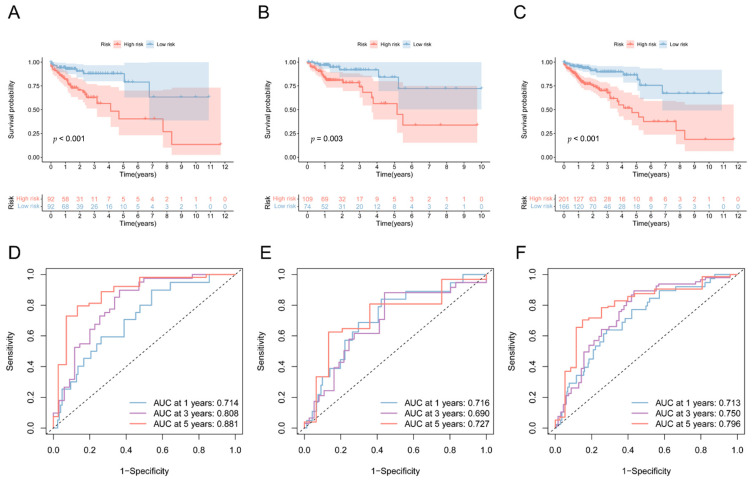
The Kaplan–Meier and ROC curve analyses for the lncRNA signature associated with 5mC. (**A**) In the training dataset, Kaplan–Meier survival curves for high-risk and low-risk subgroups. (**B**) In the testing dataset, Kaplan–Meier survival curves for high-risk and low-risk subgroups. (**C**) In the complete dataset, Kaplan–Meier survival curves for high-risk and low-risk subgroups. (**D**) The risk model’s receiver operator curve in the training dataset. (**E**) The risk model’s receiver operator curve in the testing dataset. (**F**) The risk model’s receiver operator curve in the complete dataset.

**Figure 5:**
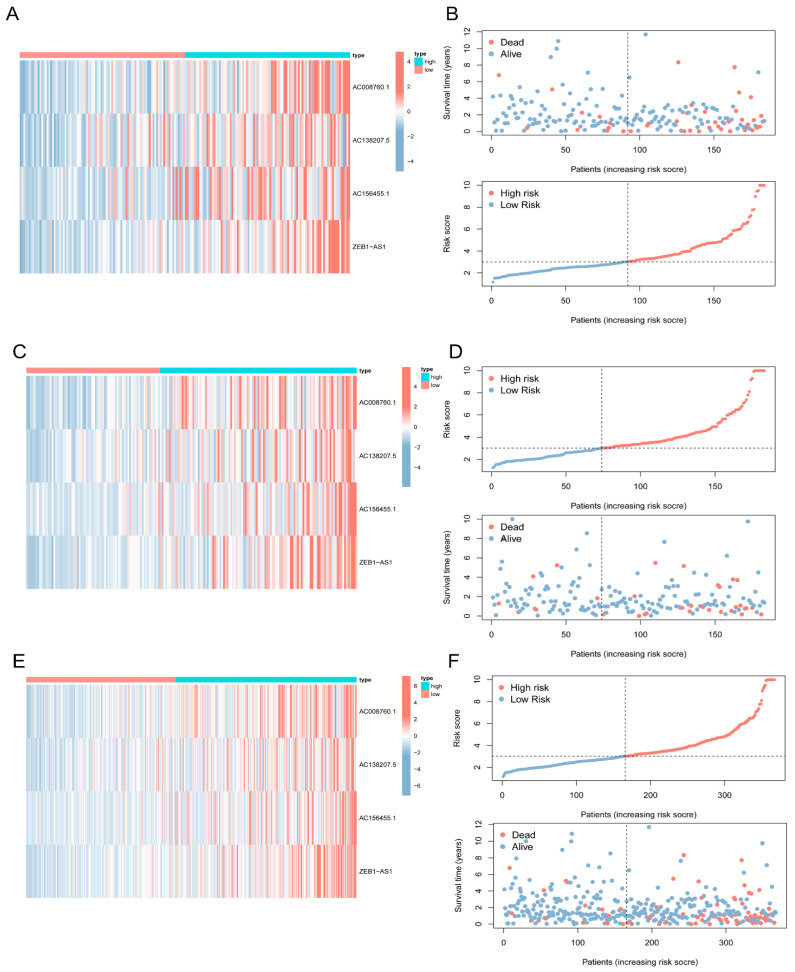
Evaluation of the lncRNA signature associated with 5mC. Heatmap revealing the expression of 4 5mC-related lncRNAs in the training dataset (**A**), testing dataset (**C**), and complete dataset (**E**), separately. Distribution of the lncRNA signature associated with 5mC risk scores and patterns of survival time and status in high-risk and low-risk subgroups in the training dataset (**B**), testing dataset (**D**), and complete dataset (**F**).

**Figure A3:**
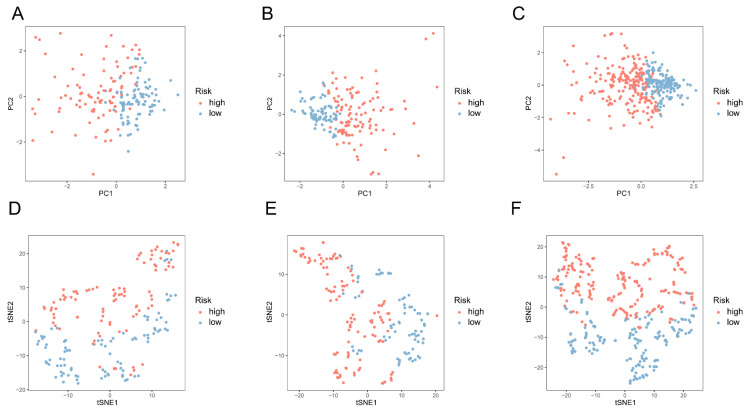
Principal component analysis and t-SNE analysis between the high- and low-risk groups in the training dataset (**A**,**D**), testing dataset (**B**,**E**), and complete dataset (**C**,**F**).

COAD patients were categorized as low-risk or high risk according to their risk score in the training dataset, testing dataset, or complete dataset. The Kaplan–Meier survival curve analysis revealed that the overall survial of COAD patients with low-risk scores was considerably longer than those of patients with high-risk scores (Figure 4A–C). The receiver operating characteristic (ROC) curves also indicated that prediction efficiency of the 4 5mC-related lncRNA prognosis signature was pretty good and was robust in predicting COAD prognosis (Figure 4D–F). The distribution of risk scores, survival status, and heatmap for the four 5mC-related lncRNAs were then evaluated. Figure 5A,C,E depicted the relative expression levels of the four 5mC-related lncRNAs in each patient. The distribution of risk classes, survival status, and survival time for each patient in each risk category is depicted in Figure 5B,D,F. Principal component analysis (PCA) revealed two markedly different distribution patterns between high-risk and low-risk groups (Figure A3A–F).

### 3.5. Prognostic Value of 5mC-Related lncRNA Prognosis Signature

**Figure 6:**
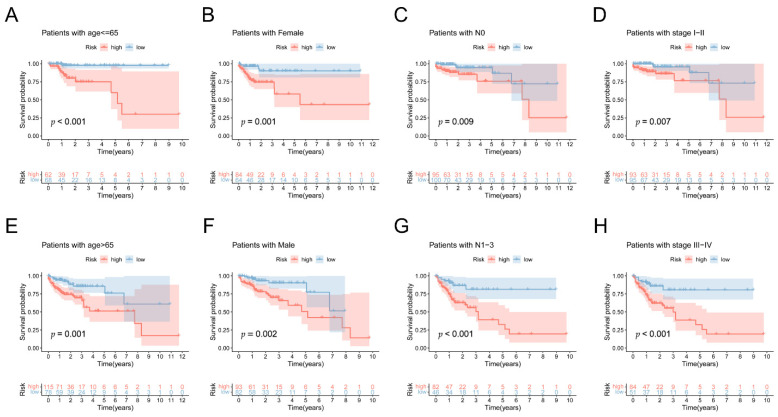
The predictive efficacy of the lncRNA signature associated with 5mC. Kaplan–Meier survival curves are classified by clinical characteristics, comprising age (**A**,**E**), gender (**B**,**F**), grade (**C**,**G**), and staging (**D**,**H**) in the complete dataset.

**Figure A4:**
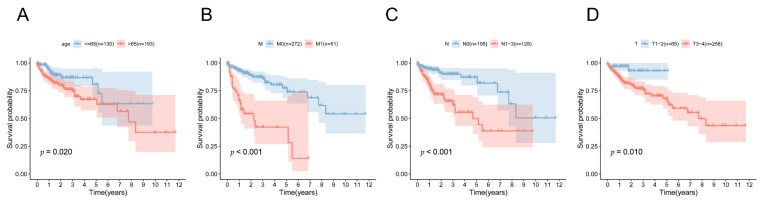
Prognostic values were evaluated according to age (**A**), T (**B**), N (**C**), and M (**D**) at the complete dataset.

**Figure A5:**
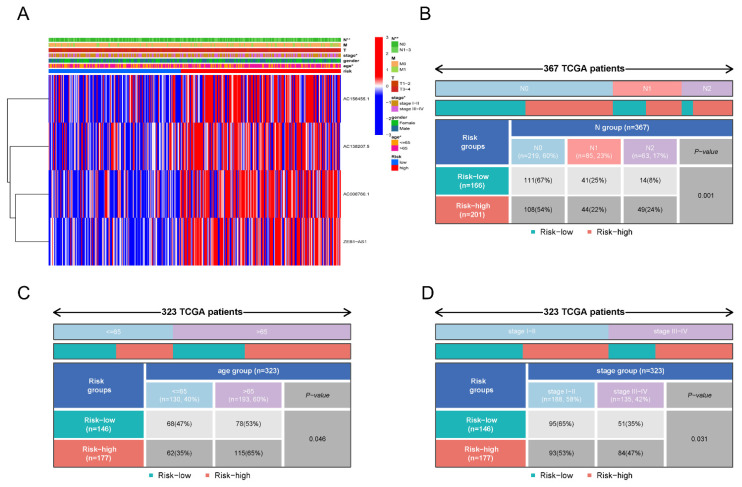
Heatmap (**A**) and table show the distribution of clinical indicators, including the N (**B**), age (**C**), and stage (**D**) between high and low risk groups. * *p* < 0.05, ** *p* < 0.01.

To evaluate the relationship between the lncRNA prognostic signature and clinical characteristics by performing Kaplan–Meier survival curve analysis, we first examined the predictive significance of the 5mC-related lncRNA signature in COAD patients. COAD patients’ independent prognostic characteristics include their age, gender, and stage. Among the subgroups, those classified as high-risk had significantly poorer survivability than those classified as low-risk (Figure 6A–H). These findings revealed that the 5mC-related lncRNA signature could be used to forecast the outcome of COAD regardless of clinical characteristics. Moreover, as illustrated in Figure A4A–D, we discovered that older individuals had a significantly higher risk score than younger individuals (*p* < 0.05). In addition, the risk score increased with the stages (*p* < 0.05). Next, we further examined the correlation between risk signature and clinicopathological characteristics. In Figure A5A–D, we found that the proportion of patients aged <65 years, N0-1, and I-II stages was almost equally distributed between the two subgroups. Nonetheless, patients aged ≥65 years, N1, and in III–VI stages were overrepresented in the high-risk subgroup compared to the low-risk subgroup. (*p* < 0.05, Chi-square test).

### 3.6. Independent Predictive Value of the 5mC-Related lncRNA Prognosis Signature

**Figure 7:**
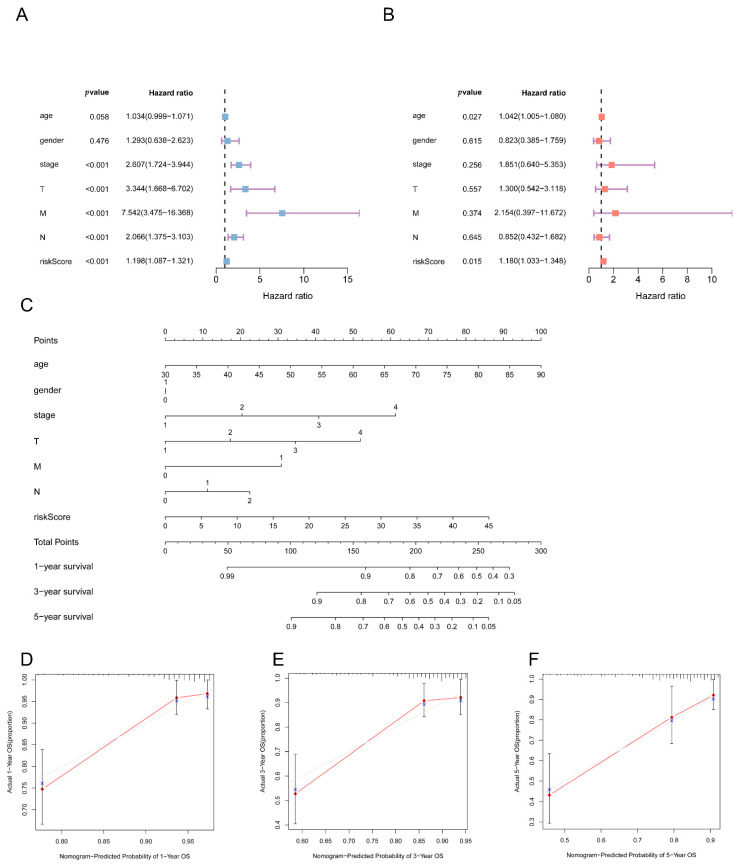
Independence and nomogram of the lncRNA signature associated with 5mC. The risk model’s independence was tested using (**A**) univariate and (**B**) multivariate Cox regression. (**C**) Based on clinical features and risk scores, a nomogram was applied to forecast the predictive capacity of overall survival in the complete dataset. The calibration plots of the nomogram for 1 (**D**), 3 (**E**), and 5 years (**F**) in the complete dataset.

To investigate the independence of the 5mC-related lncRNA prognostic signature within clinical characteristics, we performed univariate and multivariate Cox regression analyses on the training dataset. We observed that the 5mC-related lncRNA signature could be used as a stand-alone predictive biomarker for COAD (Figure 7A,B). To clarify the definition of a personalized overall survival prediction signature, we constructed a nomogram of COAD patients based on independent prognostic features (Figure 7C). The calibration plots indicated that the signature’s performance was consistent with 1-year, 3-year, and 5-year overall survival predictions (Figure 7D–F).

### 3.7. Molecular Characteristics of the 5mC-Related lncRNA Prognosis Signature

**Figure 8:**
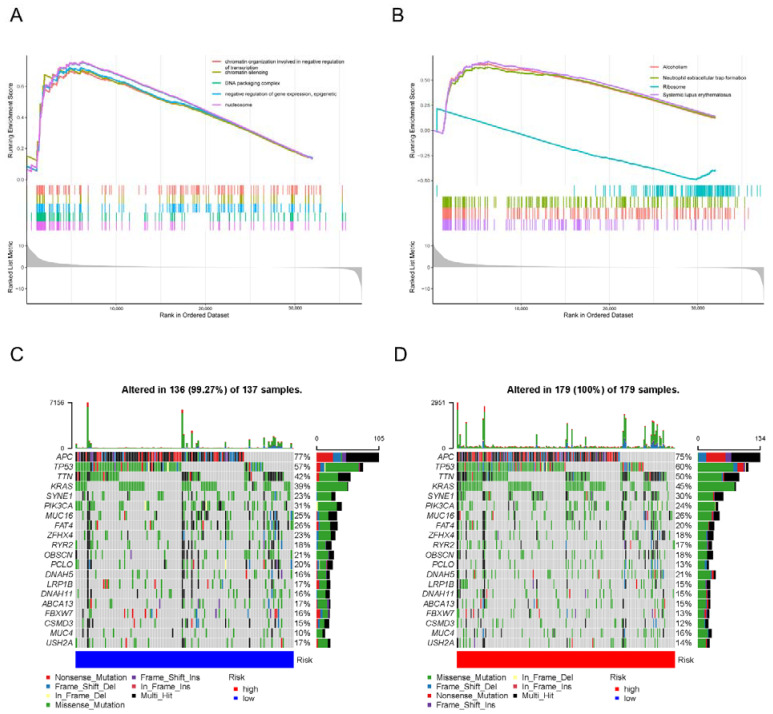
Molecular characteristics and CNV of the lncRNA signature associated with 5mC. (**A**,**B**) GSEA was utilized to analyze the molecular characteristics of various risk subgroups. (**C**) A waterfall map was applied to show the mutated genes in the COAD patients of the high-risk group. (**D**) A waterfall map was applied to show the mutated genes in the COAD patients of the low-risk group.

To get a better understanding of the biological functions and signaling pathways between the different subgroups, we conducted GSEA to identify genes that were enriched in different risk groups. As illustrated in Figure 8A,B, the high-risk group’s gene set was enriched for pathways associated with chromatin organization and systemic lupus erythematosus. Then, we further explored the differences in CNV between the risk groups, as shown in Figure 8C,D. The top 20 genes with the greatest genetic variation in risk groups were discovered, and the mutation rates of APC, TP53, TTN, and KARS were higher than 35% in both groups. Mutations in the SYNE1 gene were more common in patients classified as high-risk. While mutations in the PLCO gene were more common in patients classified as low-risk.

### 3.8. Estimation of the Tumor Immune Cell Types Utilizing the lncRNA Signature

**Figure 9:**
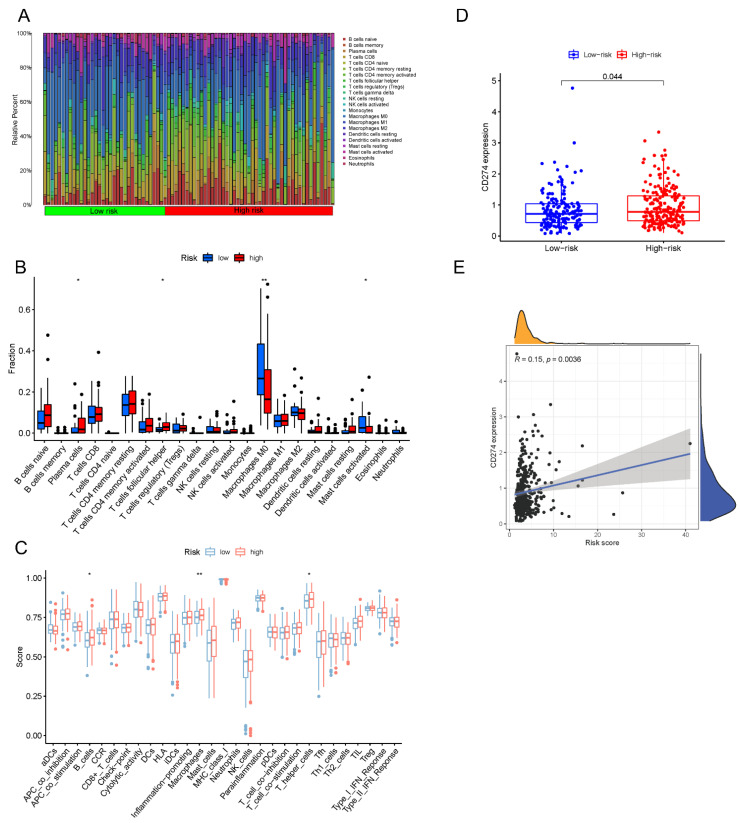
The tumor immune microenvironment and immunotherapeutic response in 5mC-related lncRNA signature. (**A**) Each patient’s relative proportion of 22 immune cells. (**B**) Comparison in immune cell expression between low- and high-risk subgroups. (**C**) Comparison in various immune functions between low- and high-risk subgroups. (**D**) Histogram showing differences in CD274 expression between low- and high-risk subgroups. (**E**) Scatter plot showing the correlation between CD274 and risk score. * *p* < 0.05, ** *p* < 0.01.

**Figure A6:**
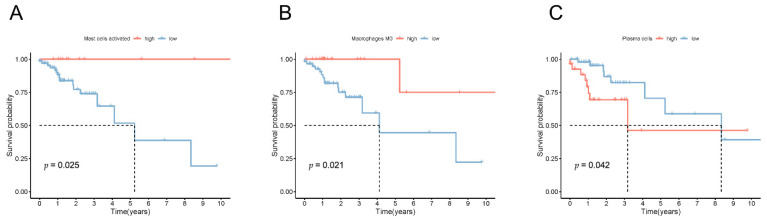
Kaplan–Meier survival curves of active mast cells (**A**), M0 macrophages (**B**), and plasma cells (**C**).

The 22 different immune cells among different risk groups were analyzed utilizing the CIBERSPRT algorithm. As a result, immune cell concentrations showed significant differences among the subgroups (Figure 9A). The risk score was subsequently shown to be positively connected with plasma and T follicular helper cells, whereas macrophages, M0 cells, and active mast cells were negatively correlated (Figure 9B). Next, survival analysis also showed dense infiltration of macrophages M0 cells and activated mast cells correlated with a better prognosis, whereas, high levels of plasma cells were associated with worse survival (Figure A6A–C). We further examined the relationships between risk factors and immunity function. According to the findings, the high-risk subgroup contained more B cells, T cells, and macrophages (Figure 9C). Given the fact that PD-L1 is a well-recognized biomarker for forecasting sensitivity to anti-PD-1/L1 therapy [37,38,39], we examined the levels of PD-L1 expression in various risk groups (Figure 9D,E). These findings indicated that PD-L1 expression was strongly related to risk score and was increased in the high-risk subgroup, which implied that the risk score could act as an indicator of immunotherapy.

## 4. Discussion

Colorectal cancer, one of the most prevalent tumors, is increasingly being investigated by scholars in terms of its development and treatment [40,41,42]. Several molecular markers have been reported as predictors of prognosis and therapeutic efficacy in colon cancer [43,44,45]. Recently, prognostic models of lncRNAs have come into the focus of researchers as predictors of prognosis and immunotherapeutic efficacy in a variety of tumors [46,47,48]. Epigenetic modifications also have gained increasing attention, such as the 5mC modifier genes, as a type of epigenetic modification, are regarded as a diagnostic and predictive indicator of malignancy [31,36]. Nevertheless, there are few reports on the significance of 5mC-related lncRNAs in the prognostic and diagnostics of COAD which deserves more investigation.

DNA 5mC methylation is a reversible post-transcriptional modification governed by 5mC-related regulators [49]. Recent research has found that 5mC genes regulate tumor growth and 5mC-regulated lncRNAs can be used as prognostic indicators of tumors [31,50,51]. In this research, we constructed a lncRNA signature containing 4 5mC-related lncRNAs to predict overall survial in COAD patients. A previous study reported that lncRNA ZEB1-AS1 drives malignant progression of COAD through the miR-455-3p/PAK2 pathway [52]. In addition, other lncRNAs were found for the first time in COAD which need to be further investigated. Following that, the Kaplan–Meier survival curve and the ROC analysis revealed that the lncRNA signature has a strong prognostic value and was highly accurate in forecasting the overall survival of COAD. We also found a significantly poorer clinical phenotype in the high-risk subgroup versus the low-risk subgroup. Additionally, we found this signature’s value at risk was an independent predictor of overall survival based on multivariate Cox regression analysis. A nomogram also showed well-consistency between observed and predicted overall survival rates at 1-, 3-, and 5-years. Our findings indicated that the lncRNA signature could serve as a potential efficient biomarker for COAD.

To assess the important biological functional phenotypes between the high-risk and low-risk groups based on the 5mC-related lncRNA prognosis signature, we conducted a GESA enrichment analysis. Nuclear lncRNAs have been known to play an important role in chromatin organization, transcription, and post-transcriptional gene expression in tumor cells [53]. Notably, the high-risk group was mainly enriched for chromatin organization, chromatin silencing, DNA packaging complex, negative regulation of gene expression, and nucleosome. Next, we studied gene mutations of different risk groups. Missense variants were the most prevalent, followed by nonsense and frameshift deletions. As previously reported, APC was the most frequently mutated gene in COAD [54], followed by TP53, TTN, and KARS. The highest mutational difference between the two groups was in the TTN mutation, which was more frequently detected in the high-risk subgroup than those from the low-risk subgroup (50% vs. 42%). The TTN gene is frequently mutated in a variety of tumor types, and frequent detection of TTN in solid tumors is related to a higher tumor mutation burden and a more favorable objective response to immune checkpoint blockade immunotherapy [55]. These findings showed that patients in the high-risk subgroup may have a better prognosis of response to immune checkpoint blockade treatment. Additionally, comparing to the high-risk subgroup, the low-risk subgroup had a higher mutation rate (20% vs. 13%) for PCLO which is frequently mutated in tumors, including hepatocellular carcinoma and diffuse large B-cell lymphoma [56,57]. Meanwhile, a mounting body of evidence suggests that mutations in PCLO are associated with tumor sensitivity to etoposide [58], implying that the sensitivity to chemotherapy may be different in various risk subgroups.

TME has been considered as a prospective biomarker for predicting response to immunotherapeutics recently [59,60]. Understanding the TME landscape of tumors may help us to find new immunotherapeutic treatments. Our results suggested that the composition of some immune cells differs within subgroups. Plasma cells and T cells follicular helper cells were significantly plentiful in the high-risk subgroup. In contrast, macrophages M0 were more common in the low-risk subgroup. Survival curves also depicted the dense infiltration of macrophages M0 cells had a better prognosis. On the contrary, dense infiltration of plasma cells had a worse prognosis. Moreover, an increasing number of studies suggested that immunotherapy with PD-L1 and PD-1 blockade is undoubtedly a breakthrough in cancer treatment [61,62]. Our results also indicated that individuals classified as high-risk have a high level of PD-L1 expression, suggesting a potential sensitivity to anti-PD-1/L1 treatment. Taken together, we conclude that this signature has strong ability to provide a good immune biomarker for COAD.

The pathological staging is still utilized to predict the prognosis in COAD patients. However, individuals with a similar stage of the disease have significantly diverse prognoses, which indicates the limitations of the current staging systems. Therefore, there is an urgent need to find better predictive and therapeutic markers to stratify patients for treatment. The 5mC-related lncRNA signature we constructed performed well in terms of COAD patient survival prediction, which could provide a new method for prognosis prediction in COAD patients. However, we also note the shortcomings and limitations of this research. Owing to the limited availability of COAD sample data (downloaded from the TCGA database: https://portal.gdc.cancer.gov, accessed on 20 September 2021), more validation to the prognostic value of the 5mC-related lncRNA signature by external clinical datasets will be needed in the next research.

## 5. Conclusions

In conclusion, we identified four 5mC-related lncRNAs that could be employed as an independent predictive indicator for COAD. This risk signature may help to differentiate between immunological and molecular features as a potential prognostic indicator for immunotherapy.

## Data Availability

Data available in a publicly accessible repository that does not issue DOIs. Publicly available datasets were analyzed in this study. This data can be found here: [https://portal.gdc.cancer.gov], accessed on 20 September 2021.
